# Variational inference for rare variant detection in deep, heterogeneous next-generation sequencing data

**DOI:** 10.1186/s12859-016-1451-5

**Published:** 2017-01-19

**Authors:** Fan Zhang, Patrick Flaherty

**Affiliations:** 1 Department of Biomedical Engineering, Worcester Polytechnic Institute, 100 Institute Road, Worcester, 01609 USA; 2 Department of Mathematics and Statistics, University of Massachusetts, Amherst, 710 N. Pleasant Street, Amherst, 01003 USA

**Keywords:** Single nucleotide variant detection, Next-generation sequencing, Bayesian statistical method, Variational inference

## Abstract

**Background:**

The detection of rare single nucleotide variants (SNVs) is important for understanding genetic heterogeneity using next-generation sequencing (NGS) data. Various computational algorithms have been proposed to detect variants at the single nucleotide level in mixed samples. Yet, the noise inherent in the biological processes involved in NGS technology necessitates the development of statistically accurate methods to identify true rare variants.

**Results:**

We propose a Bayesian statistical model and a variational expectation maximization (EM) algorithm to estimate non-reference allele frequency (NRAF) and identify SNVs in heterogeneous cell populations. We demonstrate that our variational EM algorithm has comparable sensitivity and specificity compared with a Markov Chain Monte Carlo (MCMC) sampling inference algorithm, and is more computationally efficient on tests of relatively low coverage (27× and 298×) data. Furthermore, we show that our model with a variational EM inference algorithm has higher specificity than many state-of-the-art algorithms. In an analysis of a directed evolution longitudinal yeast data set, we are able to identify a time-series trend in non-reference allele frequency and detect novel variants that have not yet been reported. Our model also detects the emergence of a beneficial variant earlier than was previously shown, and a pair of concomitant variants.

**Conclusions:**

We developed a variational EM algorithm for a hierarchical Bayesian model to identify rare variants in heterogeneous next-generation sequencing data. Our algorithm is able to identify variants in a broad range of read depths and non-reference allele frequencies with high sensitivity and specificity.

**Electronic supplementary material:**

The online version of this article (doi:10.1186/s12859-016-1451-5) contains supplementary material, which is available to authorized users.

## Background

Massively parallel sequencing data generated by next-generation sequencing technologies is routinely used to interrogate single nucleotide variants (SNVs) in research samples [1]. For example, deep sequencing confirmed the degree of genetic heterogeneity of HIV and influenza [2, 3]. Intra-tumor heterogeneity has been revealed by next-generation sequencing [4]. Whole genome sequencing has revealed that many beneficial mutations of minor allele frequencies are essential to respond to dynamic environments [5]. However, rare SNV identification in heterogeneous cell populations is challenging, because of the intrinsic error rate of next generation sequencing [6]. Thus, there is a need for accurate and scalable statistical methods to uncover SNVs in heterogeneous samples.

A number of computational methods have been developed to detect SNVs in large scale genomic data sets. These methods can be roughly categorized as probabilistic or heuristic or some combination. Among all of the current probabilistic methods, the Bayesian probabilistic framework has been increasingly used to estimate unobserved quantities such as variant allele frequency given observed genomic sequencing data.

GATK [7] and SAMTools [8] use a naive Bayesian decision rule to call variants. EBCall models sequencing errors based on a Beta-Binomial distribution, where the parameters and latent variables are estimated from a set of non-paired normal sequencing samples [9]. However, the error rate of normal sequencing samples could be unmatched with the error rate of the target samples, which may cause a problem of making false negatives calls [10]. CRISP compares aligned reads across multiple pools to obtain sequencing errors, and then distinguishes true rare variants from the sequencing errors [11]. However, the bottleneck of CRISP is its low computational efficiency due to a calculation of a large number of contingency tables.

JointSNVMix introduces two Bayesian probabilistic models (JointSNVMix1 and JointSNVMix2) to jointly analyze a tumour-normal paired allelic count of NGS data [12]. JointSNVMix derives an expectation maximization (EM) algorithm to calculate maximum a-posteriori (MAP) estimate of latent variables in a particular probabilistic graphical model. Furthermore, they showed that the joint modeling method, JointSNVMix1, observes 80-fold reduction of false positives compared with its independent analogue (SNVMix1) [12]. SomaticSniper models the joint diploid genotype likelihoods for both tumour and normal samples [13]. Strelka models the joint probabilistic distribution of allele frequencies for both tumour and normal samples, which is demonstrated to be more accurate compared with the methods based on the estimated allele frequency tests between tumour and normal samples [14]. SNVer focuses on a frequentist method that is able to calculate *P*-values, but [15] pointed out that this approach fails to model sampling bias that will reduce the power of detecting true rare variants. VarScan compares tumour and normal samples thresholding on variant allele frequency and a number of allele counts then uses Fisher’s exact test to estimate sample allele frequencies [16].

In previous work, we developed a Beta-Binomial model to estimate a null hypothesis error rate distribution at each position. Using this rare variant detection (RVD) model, we call rare variants by comparing the error rate of the sample sequence data to a null distribution obtained from sequencing a known reference sample [2]. RVD can identify mutant positions at a 0.1% fraction in mixed samples using high read depth data.

An improvement of that work, RVD2, uses hierarchical priors to tie parameters across positions to detect variants in low read depth data [17]. We derived a Markov Chain Monte Carlo (MCMC) sampling algorithm for posterior inference. However, the main limitation of MCMC is that it is hard to diagnose convergence and may be slow to converge [18]. An alternative inference method, that we explore here, is to use variational inference, which is based on a proposed variational distribution over latent variables. By optimizing variational parameters, we fit an approximate distribution that is close to the true posterior distribution in the sense of the Kullback-Liebler (KL) divergence. Variational inference can now handle nonconjugate distributions and tends to be more computationally efficient than MCMC sampling [19].

Here, we propose a variational EM algorithm for our Bayesian statistical model to detect rare SNVs in heterogeneous NGS data. We show that variational EM algorithm has comparable accuracy and efficiency compared with MCMC in a synthetic data set. First, we define the model structure, and derive our variational EM algorithm to approximate the posterior distribution over latent variables. Then, we call a variant by a posterior difference hypothesis test between the key model parameters of a pair of samples. As a result, we compare the performance of the variational EM inference algorithm to the MCMC sampling method and the state-of-the-art methods using a synthetic data set. Finally, we show that our variational EM algorithm is able to detect rare variants and estimate non-reference allele frequency (NRAF) in a longitudinal directed evolution experimental data set.

## Methods

### Model structure

**Fig. 1:**
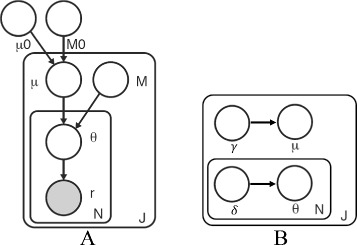
Graphical model. **a** Graphical model representation of the model. **b** Graphical model representation of the variational approximation to approximate the posterior distribution. Observed random variables are shown as shaded nodes and latent random variables are unshaded. The object of inference for the variational EM algorithm is the joint distribution *p*(*μ*,*θ*|*r*,*n*)

Our Bayesian statistical model is shown as a graphical model in Fig. 1 a. In the model, *r*_*ji*_ is the number of reads with a non-reference base at location *j* in experimental replicate *i*; *n*_*ji*_ is the total number of reads at location *j* in experimental replicate *i*. The model parameters are: −*μ*_0_ a global non-reference read rate that captures the error rate across all the positions, − *M*_0_ a global precision that captures the variation of the error rate across positions in a sequence, and − *M*_*j*_ a local precision that captures the variation of the error rate at position *j* across different replicates.

The latent variables are: − *μ*_*j*_∼Beta(*μ*_0_,*M*_0_) a position-specific non-reference read rate for position *j*, and − *θ*_*ji*_∼Beta(*μ*_*j*_,*M*_*j*_) the non-reference read rate for position *j* in replicate *i*.

In Fig. 1 b, *γ* is the parameter for the variational distribution for latent variable *μ*, and *δ* is the parameter for the variational distribution for latent variable *θ*. We describe *q*(*μ*) and *q*(*θ*) in detail in the following section.

The model generative process is as follows:

1. For each location *j*∈ [ 1,…,*J*]:
  - Draw an error rate $\mu _{j} \thicksim \text {Beta}(\mu _{0}, M_{0})$
  - For each replicate *i*∈ [ 1,…,*N*]:
    (i) Draw $\theta _{ji} \thicksim \text {Beta}(\mu _{j}, M_{j})$
    (ii) Draw $r_{ji} | n_{ji} \thicksim \text {Binomial}(\theta _{ji}, n_{ji})$

The joint distribution *p*(*r*,*μ*,*θ*|*n*;*ϕ*) given the parameters can be factorized as

1 $$ p(r, \mu, \theta| n; \phi) = p(r |\theta, n)p(\theta |\mu; M)p(\mu;\mu_{0}, M_{0}).  $$

### Variational expectation maximization (EM) inference

We developed a non-conjugate variational inference algorithm to approximate the posterior distribution,

2 $$ p(\mu, \theta | r, n; \phi) = \frac{p(r, \mu, \theta| n; \phi)} {p (r | n; \phi)},  $$

where the parameters are $\phi \triangleq \{\mu _{0}, M_{0}, M\}$.

#### Factorization

We propose the following factorized variational distribution to approximate the true posterior over latent variables *μ*_*j*_ and *θ*_*ji*_. Here, *q*(*μ*_*j*_) approximates the variational posterior distribution of *μ*_*j*_, which represents the local error rate distribution at position *j* across different replicates; and *q*(*θ*_*ji*_) approximates the posterior distribution of *θ*_*ji*_, which is the error rate distribution at position *j* for replicate *i*.

3 $$ q(\mu, \theta) = q(\mu)q(\theta) = \prod_{j=1}^{J} q(\mu_{j}) \prod_{i=1}^{N} q(\theta_{ji}).   $$

#### Evidence lower bound (ELBO)

Given the variational distribution, *q*, the log-likelihood of the data is lower-bounded according to Jensen’s inequality,

4 $${} \begin{array}{ll} \log p \left(r | n; \phi \right) &= \log \int_{\mu} \int_{\theta} p\left(r,\mu,\theta |n; \phi \right) d\theta d\mu \\ &= \log \int_{\mu} \int_{\theta} p\left(r,\mu,\theta |n; \phi \right)\frac{q\left(\mu,\theta \right) }{q\left(\mu,\theta \right)} d\theta d\mu \\ &\geq \int_{\mu} \int_{\theta} q\left(\mu,\theta \right) \log \frac{p\left(r,\mu,\theta |n; \phi \right)}{q\left(\mu,\theta \right)} d\theta d\mu \\ &= E_{q} \left[ \log p\left(r,\mu,\theta |n; \phi \right)\right] - E_{q} \left[ \log q\left(\mu,\theta \right)\right] \\ &\triangleq \mathcal{L}(q, \phi). \end{array}  $$

The function $\mathcal {L}(q, \phi)$ is the evidence of lower bound (ELBO) of the log-likelihood of the data, which is the sum of *q*-expected complete log-likelihood and the entropy of the variational distribution *q*. The goal of variational inference is to maximize the ELBO. Equivalently, *q* is chosen by minimizing the KL divergence between the variational distribution and the true posterior distribution.

Since *θ* and *r* are conjugate pairs, the posterior distribution of *θ*_*ji*_ is a Beta distribution,

5 $$\begin{array}{*{20}l}{} &p(\theta_{ji}|r_{ji},n_{ji},\mu_{j},M_{j}) \!\thicksim \text{\!Beta}(r_{ji}\,+\,M_{j} \mu_{j}, n_{ji}-r_{ji}\,+\,M_{j}(1\,-\,\mu_{j})). \end{array} $$

Therefore, we propose a Beta distribution with parameter vector *δ*_*ji*_ as variational distribution,

$$\begin{array}{*{20}l} \theta_{ji} &\thicksim \text{Beta}(\delta_{ji1}, \delta_{ji2}). \end{array} $$

The posterior distribution of *μ*_*j*_ is given by its Markov blanket,

6 $$\begin{array}{*{20}l} p(\mu_{j}|\theta_{ji},M_{j},\mu_{0},M_{0})\propto p(\mu_{j}|\mu_{0},M_{0})p(\theta_{ji}|\mu_{j},M_{j}). \end{array} $$

This is not in the form of any known distribution. But, since the support of *μ*_*j*_ is [ 0,1], we propose a Beta distribution with parameter vector *γ*_*j*_ as variational distribution,

$$\begin{array}{*{20}l} \mu_{j} &\thicksim \text{Beta}(\gamma_{j1}, \gamma_{j2}). \end{array} $$

Each component of ELBO is derived in Additional file 1.

#### Variational EM algorithm

Variational EM algorithm maximizes the ELBO of the likelihood by alternating between maximization over *q* (E-step) and maximization over *ϕ*={*μ*_0_,*M*_0_,*M*} (M-step). We update the variational parameters and the model parameters iteratively by numerically optimizing each problem using Sequential Least SQuares Programming (SLSQP) [20] (see Additional file 2 for detail). There is no analytical representation for $E_{q}\left [ \log \left (\frac {\Gamma (M_{j})} { \Gamma (\mu _{j} M_{j}) \Gamma (M_{j} (1-\mu _{j})) }\right)\right ]$, which is required to update variational distribution for *μ*_*j*_ and model parameter *M*. So, we must resort to numerical integration,

7 $$ \begin{array}{ll} & E_{q}\left[ \log \left(\frac{\Gamma(M_{j})} { \Gamma(\mu_{j} M_{j}) \Gamma((1-\mu_{j})M_{j}) }\right)\right] =\\ & \int_{0}^{1} q(\mu_{j};\gamma_{j1}, \gamma_{j2}) \log \left(\frac{\Gamma(M_{j})} { \Gamma(\mu_{j} M_{j}) \Gamma((1-\mu_{j})M_{j}) }\right) d\mu_{j}, \end{array}  $$

Unfortunately, this numerical integration step is computationally expensive. The variational EM algorithm is summarized using pseudocode in Algorithm 1.



### Hypothesis testing

The posterior distribution over $\mu _{j}^{\triangle } \mid r^{case}, r^{control} \triangleq \mu _{j}|r^{case} - \mu _{j}|r^{control}$ is the distribution over the change in the non-reference read rate at position *j* between a case and control sample. Since the variational approximate posterior distributions in the difference are Beta distributions, the distribution of the difference is not analytically known. In order to compute the statistic of interest, we approximate *μ*_*j*_|*r* ^*case*^ and *μ*_*j*_|*r* ^*control*^ with univariate Gaussian distributions by matching the first two moments of the variational Beta distributions. Then, the difference is a Gaussian distribution. As we show in the section of comparison of approximated posterior distribution, the Gaussian approximation is empirically reasonable.

Under the variational approximation,

8 $$\begin{array}{*{20}l} E_{q}[\mu_{j}|r^{case}] &= \frac{\gamma_{j1}^{case}}{\gamma_{j1}^{case} + \gamma_{j2}^{case}} \end{array} $$

9 $$\begin{array}{*{20}l} \text{Var}_{q}[\mu_{j}|r^{case}] &= \frac{\gamma_{j1}^{case} \gamma_{j2}^{case}}{(\gamma_{j1}^{case} + \gamma_{j2}^{case} + 1)(\gamma_{j1}^{case} + \gamma_{j2}^{case})^{2}} \end{array} $$

for *μ*_*j*_|*r* ^*case*^ and likewise for *μ*_*j*_|*r* ^*control*^. We approximate the posterior for the case sample as

10 $$ \mu_{j} | r^{case} \sim \mathcal{N}(E_{q}[\mu_{j}|r^{case}], \text{Var}_{q}[\mu_{j}|r^{case}])  $$

and likewise for the control. Then,

11 $${} \begin{array}{ll} &\mu_{j}^{\triangle} \mid r^{case}, r^{control} \sim \\ &\quad \mathcal{N}(E_{q}[\mu_{j}|r^{case}] - E_{q}[\mu_{j}|r^{control}], \text{Var}_{q}[\mu_{j}|r^{case}]\\ &\quad+ \text{Var}_{q}[\mu_{j}|r^{control}]) \end{array}  $$

Now, we can approximate the posterior probability of interest,

12 $$ \Pr(\mu_{j}^{\triangle} \geq \tau \mid r^{case}, r^{control}),  $$

that is, the posterior probability that the difference in the non-reference read rate is greater than a fixed effect size *τ* (e.g. zero) for a one sided test. For a two sided test, we compute the approximate probability

13 $$ \Pr(| \mu_{j}^{\triangle} | \geq \tau \mid r^{case}, r^{control}).  $$

A position is called a *provisional variant* if $\Pr (| \mu _{j}^{\triangle } | \geq \tau \mid r^{case}, r^{control}) \geq 1-\alpha /2$, where the probability is approximated as described.

It is possible that a position is called a variant due to a differential non-reference read count, but no particular alternative base is more frequently observed than the others. In this case, the likely cause is a sequencing error that indiscriminately incorporates a non-reference base at the position. To discriminate this non-biological cause from the interesting true variants we use a *χ* ^2^ goodness-of-fit test for non-uniform base distribution [17, 21]. For each provisional variant, if we reject the null hypothesis that the distribution is uniform, we promote the position to a *called variant*.

## Results

### Data sets

#### Synthetic DNA sequence data

The data set we use to assess sensitivity and specificity is described and made available elsewhere [2]. Briefly, we performed an in-vitro mixture of two DNA sequences to test the sensitivity and specificity of our approach. Two 400 bp DNA sequences were chemically synthesized. One sample has 14 variant loci and is taken as the case and the other without variants is taken as the control. Case and control DNA samples were mixed in-vitro to yield defined NRAF of 0.1%, 0.3%, 1.0%, 10.0%, and 100.0%. The synthetic DNA dataset was downsampled by 10×, 100×, 1,000×, and 10,000× using picard (v 1.96). The final data set contains read pairs for six replicates for the control and cases at different NRAF levels.

#### Longitudinal directed evolution data

The longitudinal yeast data comes from three strains of haploid S288c which were grown for 448 generations under limited-glucose (0.08%). The wild-type ancestral strain GSY1136 was sequenced as a reference. Aliquots were taken about every 70 generations and sequenced. The detail of library sequencing is described in [5, 11, 22]. The Illumina sequencing data is available on the NCBI Sequence Read Archive (SRA054922)[5]. For this study, we received the original BAM files from one of the authors. The aligned BAM files have 266 – 1,046× coverage. We used samtools (v 1.1) with -mpileup -C50 flags to convert BAM files to pileup files. Then, we generated depth chart files, which are tab-delimited text tables recording in each element of the table the count of a nucleotide at a genomic position. We ran our variational inference algorithm on the depth chart files to identify SNVs.

### Performance on synthetic DNA data

#### Comparison of sensitivity and specificity

**Fig. 2:**
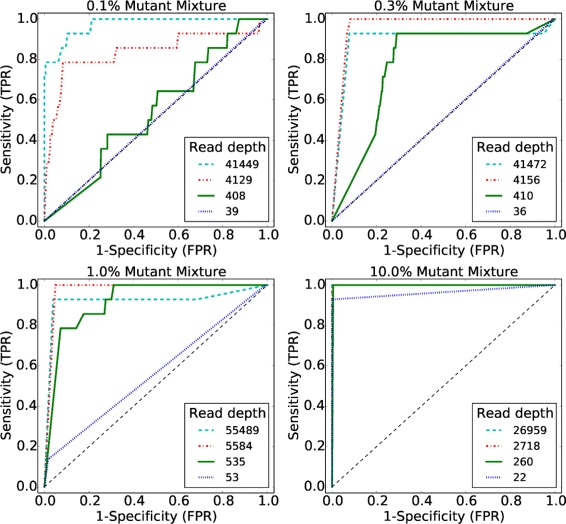
ROC curves with varying median read depths and NRAFs

The performance of variational EM algorithm is shown in receiver-operating characteristic curves (ROCs) for a broad range of median read depths and NRAFs in Fig. 2. The results in the ROC curves are generated by varying parameter *α* in the posterior distribution test. It shows that the performance improved with read depth and true mutant mixtures. Furthermore, we evaluated the performance by using both the posterior distribution test with *α*=0.05 and the *χ* ^2^ test to detect variants, and compared the performance with the MCMC sampling algorithm in terms of sensitivity and specificity (Table 1).

**Table 1:** Sensitivity/Specificity comparison of variational EM algorithm with MCMC algorithm

True NRAF	Median depth	Sensitivity		Specificity	
		MCMC	Variational	MCMC	Variational
0.1%	39	0.00	0.00	1.00	1.00
	408	0.00	0.07	1.00	1.00
	4129	0.14	0.29	1.00	1.00
	41449	0.86	1.00	0.97	1.00
0.3%	36	0.00	0.00	1.00	1.00
	410	0.00	0.00	1.00	1.00
	4156	1.00	1.00	0.99	0.98
	41472	1.00	0.93	0.85	0.91
1.0%	53	0.00	0.00	1.00	1.00
	535	0.21	0.29	1.00	1.00
	5584	1.00	1.00	0.98	0.98
	55489	1.00	0.93	0.87	0.95
10.0%	22	0.00	0.57	1.00	1.00
	260	1.00	1.00	1.00	1.00
	2718	1.00	1.00	1.00	1.00
	26959	1.00	1.00	1.00	1.00
100.0%	27	1.00	1.00	1.00	1.00
	298	1.00	1.00	1.00	1.00
	3089	1.00	1.00	1.00	1.00
	30590	1.00	1.00	1.00	1.00

The variational EM algorithm shows higher sensitivity and specificity than the MCMC algorithm in the events when NRAF is 0.1%. The variational EM algorithm has a higher specificity compared with the MCMC algorithm for a median read depth of 41,472× at 0.3% NRAF and 55,489× at 1.0% NRAF, but the sensitivity is slightly lower due to false negatives.

#### Comparison of approximated posterior distribution

**Fig. 3:**
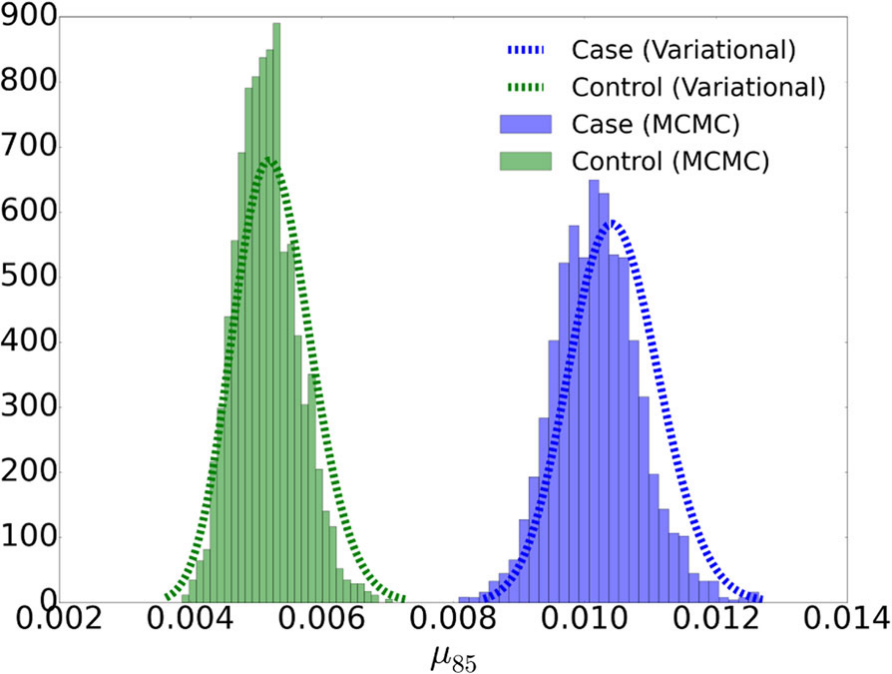
Approximated posterior distributions by the variational EM and MCMC algorithms for a true variant position 85 when the median read depth is 5,584×

Figure 3 shows the approximate posterior distribution of the variational EM algorithm and samples of the MCMC algorithm. One variant position, 85, is taken as an example to show the comparison of the approximated posteriors. The variational EM and MCMC algorithms both identify all the variants when NRAF is 10.0% and 100.0%. The variational EM algorithm calls 90 false positive positions without a *χ* ^2^ test when NRAFs are 0.1% and 0.3% for low median read depth (30× and 400×). This is to be expected because it is highly unlikely to correctly identify a variant base with a population frequency of 1 in 1,000 with less than a 1,000× read depth.

**Fig. 4:**
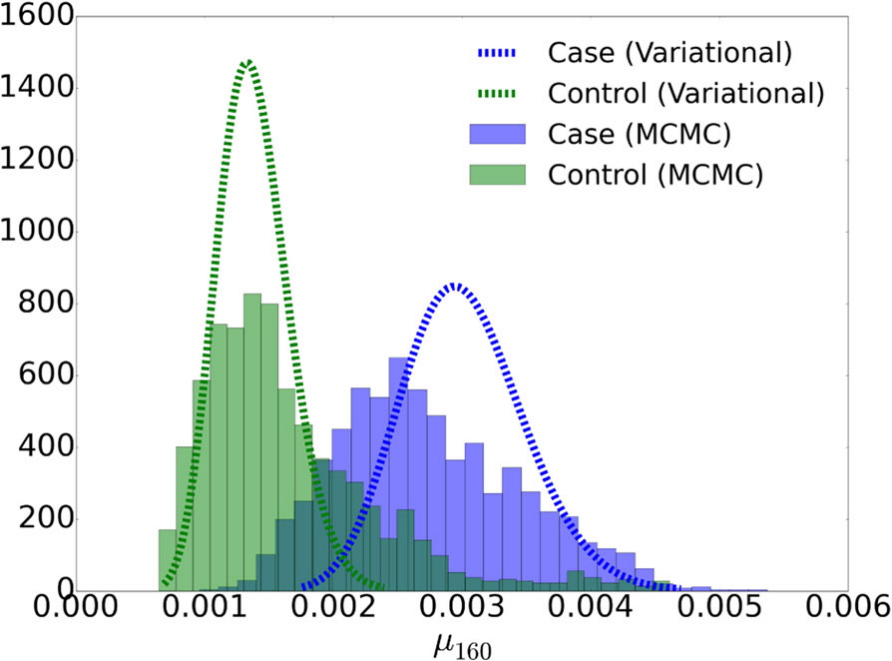
Approximated posterior distribution by the variational EM and MCMC algorithms for a non-variant position (160) that was not called by the MCMC algorithm (true negative), but was called by the variational EM algorithm (false positive) with a median read depth of 410×

A false positive, a non-mutated position that is called by the variational EM algorithm but not called by the MCMC algorithm, is shown in Fig. 4. The variance of the MCMC posterior estimate is higher than that of the variational posterior estimate. We tested 10 random initial values variational inference algorithm and found the approximate posterior distributions from the variational EM algorithm are essentially equivalent for all random initializations. It is notable that the shape of the proposed Beta variational distribution is well approximated by a Gaussian.

#### Comparison to the state-of-the-art methods

We compared the performance of our variational EM algorithm with the state-of-the-art variant detection methods, SAMtools [8], GATK [7], CRISP [11], VarScan2 [16], Strelka [14], SNVer [15], MuTect [23], and RVD2 [17], using synthetic DNA data set (Table 2). Among all of the methods compared, our variational EM algorithm has a higher sensitivity and specificity for a broad range of read depths and NRAFs. Our variational EM algorithm shows higher specificity than all the other tested methods at a very low NRAF (0.1%) level. However, our algorithm has a slightly lower specificity than the MCMC algorithm when the median read depth is 4,156× at 0.3% NRAF, and a slightly lower sensitivity than the MCMC algorithm when the median read depth is 41,472× at 0.3% NRAF and a median read depth of 55,489× at 1.0% NRAF. The performance of other methods is stated in detail in [17].

**Table 2:** Sensitivity/Specificity comparison with other variant detection methods

NRAF	Median	SAMtools	GATK	CRISP	VarScan2	VarScan2	Strelka	SNVer	MuTect	RVD2	RVD2
	depth				mpileup	somatic				MCMC	Variational
0.1%	39	0.00/1.00	0.00/1.00	0.00/1.00	0.00/1.00	0.00/1.00	0.00/1.00	0.00/1.00	0.00/0.99	0.00/1.00	0.00/1.00
	408	0.00/1.00	0.00/1.00	0.00/1.00	0.00/1.00	0.07/0.92	0.00/1.00	0.00/1.00	0.29/0.91	0.00/1.00	0.07/1.00
	4129	0.00/1.00	0.00/1.00	0.00/1.00	0.00/1.00	0.57/0.52	0.00/1.00	0.00/1.00	0.64/0.86	0.14/1.00	0.29/1.00
	41449	0.00/1.00	0.00/1.00	0.00/1.00	0.00/1.00	0.64/0.79	0.00/1.00	0.00/1.00	0.14/0.93	0.86/0.97	1.00/1.00
0.3%	36	0.00/1.00	0.00/1.00	0.00/1.00	0.00/1.00	0.00/1.00	0.00/1.00	0.00/1.00	0.43/1.00	0.00/1.00	0.00/1.00
	410	0.00/1.00	0.00/1.00	0.00/1.00	0.00/1.00	0.21/0.95	0.00/1.00	0.00/1.00	0.50/0.94	0.00/1.00	0.00/1.00
	4156	0.00/1.00	0.00/1.00	0.00/1.00	0.00/1.00	0.57/0.53	0.00/1.00	0.21/0.99	0.36/0.91	1.00/0.99	1.00/0.98
	41472	0.00/1.00	0.00/1.00	0.00/1.00	0.00/1.00	0.64/0.75	0.00/1.00	0.86/0.97	0.43/0.90	1.00/0.85	0.93/0.91
1.0%	53	0.00/1.00	0.00/1.00	0.00/1.00	0.00/1.00	0.00/0.99	0.00/1.00	0.00/1.00	0.29/0.98	0.00/1.00	0.00/1.00
	535	0.00/1.00	0.00/1.00	0.00/1.00	0.00/1.00	0.43/0.89	0.00/1.00	0.29/1.00	0.71/0.91	0.21/1.00	0.29/1.00
	5584	0.00/1.00	0.00/1.00	0.00/1.00	0.00/1.00	0.57/0.47	0.00/1.00	1.00/0.99	0.64/0.95	1.00/0.98	1.00/0.98
	55489	0.00/1.00	0.00/1.00	0.00/1.00	0.00/1.00	0.64/0.69	0.00/1.00	1.00/0.97	0.86/0.90	1.00/0.87	0.93/0.95
10.0%	22	0.21/1.00	0.43/1.00	0.86/1.00	0.00/1.00	0.36/1.00	0.29/1.00	0.93/1.00	0.86/0.99	0.00/1.00	0.57/1.00
	260	0.00/1.00	0.57/1.00	1.00/1.00	0.00/1.00	0.86/1.00	1.00/1.00	1.00/1.00	1.00/0.99	1.00/1.00	1.00/1.00
	2718	0.00/1.00	0.79/1.00	0.07/1.00	0.00/1.00	0.57/0.78	1.00/1.00	1.00/0.99	1.00/0.98	1.00/1.00	1.00/1.00
	26959	0.00/1.00	0.57/1.00	0.00/1.00	0.00/1.00	0.64/0.53	1.00/0.99	1.00/0.98	1.00/0.98	1.00/1.00	1.00/1.00
100.0%	27	1.00/0.99	1.00/1.00	1.00/1.00	1.00/1.00	1.00/1.00	1.00/1.00	1.00/0.99	1.00/0.98	1.00/1.00	1.00/1.00
	298	1.00/0.99	1.00/1.00	1.00/1.00	1.00/1.00	1.00/0.99	1.00/0.99	1.00/0.98	1.00/0.98	1.00/1.00	1.00/1.00
	3089	0.86/1.00	1.00/1.00	1.00/0.99	1.00/1.00	1.00/0.65	1.00/0.99	1.00/0.98	1.00/0.98	1.00/1.00	1.00/1.00
	30590	0.71/1.00	1.00/1.00	1.00/1.00	1.00/1.00	1.00/0.39	1.00/1.00	1.00/0.98	1.00/0.99	1.00/1.00	1.00/1.00

#### Runtime assessment

**Fig. 5:**
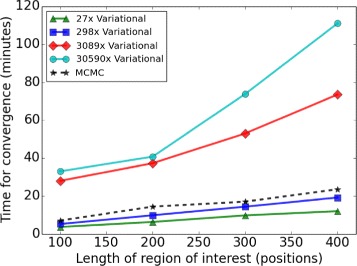
Computational efficiency comparison for our variational EM algorithm and MCMC sampling algorithm. Sixty processors are used to estimate the model on the synthetic data set

The computational time for approximating the variational posterior distribution is increased by expanding the length of region and the median read depth (Fig. 5). Our variational EM algorithm is faster than the MCMC algorithm at the low median read depths of 27× and 298×, and slower for the high median read depths of 3,089× and 30,590×.

Table 3 shows the timing profile for each part of our variational EM algorithm when median read depth is 3,089×. Optimizing *γ* in the E-step and optimizing *M*_*j*_ in the M-step takes more than 95% of the time of one variational iteration in a test of a single processor, since the integration (7) is needed.

**Table 3:** Timing profile of variational EM algorithm when median depth is 3,089×

		E-step			M-step				
Computation	Region	Optimize	Optimize	Update	Optimize	Optimize	Optimize	Update	Total
resource	length	*γ*	*δ*	ELBO	*μ*_0_	*M*_0_	*M*	ELBO	time (s)
Single processor	100	617.7 (63%)	4.232	10.42	0.264	0.159	332.8 (34%)	10.29	976.0
	200	1124 (65%)	8.936	18.64	0.418	0.256	570.0 (33%)	18.37	1741
	300	1728 (65%)	13.27	27.81	0.445	0.400	851.5 (32%)	27.65	2649
	400	2433 (66%)	17.99	38.55	0.737	0.635	1176 (32%)	38.17	3705
60 processors	100	29.93 (41%)	0.2470	11.67	0.3070	0.1890	19.56 (26%)	11.98	73.89
	200	44.69 (40%)	0.4170	22.14	0.5230	0.3040	24.04 (21%)	22.24	114.3
	300	63.47 (40%)	0.7160	33.31	0.5620	0.5040	29.41 (18%)	33.24	161.2
	400	94.66 (43%)	0.7270	42.78	0.8200	0.7060	40.04 (18%)	44.28	219.7

Timing profile of 4 significant figures for one iteration of variational EM algorithm when median read depth is 3,089×. Single and multiple processors are both tested to estimate computational efficiency. Time for optimizing *γ* in the E-step and optimizing *M* in the M-step is highlighted in percentage

### Variant detection on the longitudinal directed evolution data

#### Detected variants

We applied our variational EM algorithm to the MTH1 gene at Chr04:1,014,401-1,015,702 (1,302 bp), which is the most frequently observed mutated gene by [5]. Our algorithm detected the same variants that were found by [5] (shown as highlighted in Additional file 2). Additionally, we detected 81 novel variants in 8 timepoints that the original publication did not detect. In Additional file 2, G7 is the baseline NRAF as the control sample when comparing with G70, G133, G266, G322, G385, and G448 in the respective hypotheses testing. The corresponding NRAFs of called variants at different time points are given by the estimate of the latent variable, $\hat {\mu _{j}} = E_{q}[\mu _{j}|r]$.

All of these variants, except the variant at position Chr04:1,014,740, decrease in NRAF following a maximum. The allele at position Chr04:1,014,740 is a beneficial variant that arises in NRAF to 99.6% at generation 448 within a constant glucose-limited environment. Moreover, we identified the first emergence of this beneficial variant as early as 0.5% in generation 133. We detected 22 variants (NRAF < 1.0%) early (at generation 70) in the evolutionary time course. Given that the median read depth is 1,649×, we have some confidence these are bona-fide variants.

#### Concomitant variants detection

**Fig. 6:**
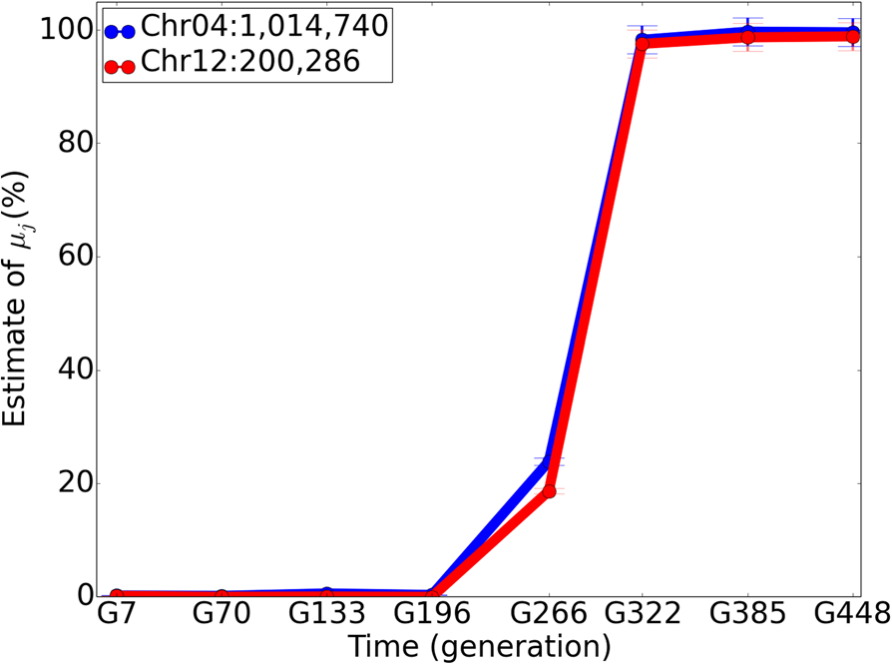
The NRAF trend of concomitant variants in gene MTH1 and ADE16. The 95% Bayesian credible intervals are shown

We identified a pair of variants, Chr04:1,014,740 in gene MTH1 and Chr12:200,286 in gene ADE16, that increase in NRAF together in time (Fig. 6). We hypotheses that the variants are concomitant in the same clone. In this pair of genes, gene MTH1 is a negative regulator of the glucose-sensing signal transduction pathway, and gene ADE16 is an enzyme of *d* *e* *n* *o* *v* *o* purine biosynthesis. Glucose sensing induces gene expression changes to help yeast receive necessary nutrients, which could be a reason for this pair of genes to mutate together [24]. Further experimental validation of this hypothesis would be required to definitively show that the mutations are concomitant.

## Discussion

### Sensitivity analysis

**Fig. 7:**
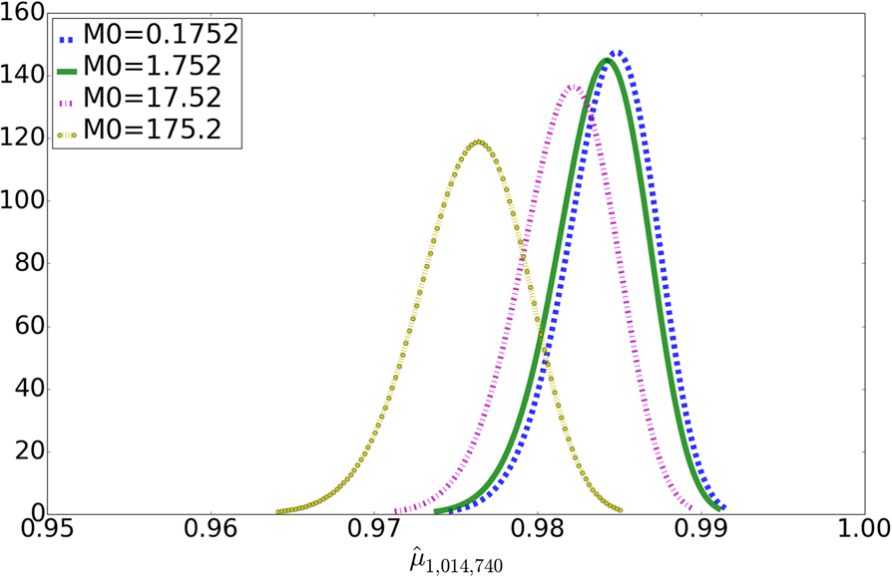
Influence of *M*_0_ on the estimate of *μ*_*j*_. Posterior distributions of the variant at position Chr04:1,014,740, $\hat {\mu }_{1,014,740}$, with different $\hat {M_{0}}$ are shown

The global precision hyper-parameter *M*_0_ could influence the estimate of *μ*_*j*_ due to its regularization effect. We show the influence of different $\hat {M_{0}}$ on variant position Chr04:1,014,740, *q*(*μ*_1,014,740_|*r*) in Fig. 7. We see that as we decrease the prior precision parameter $\hat {M_{0}}$, $\hat {\mu }_{1,014,740}$ increases as expected. But the effect of changing $\hat {M_{0}}$ over several orders of magnitude does not change $\hat {\mu }_{j}$ greatly. Here $\hat {M_{0}} = 1.752$ in this dataset.

## Conclusions

In this article, we propose a variational EM algorithm to estimate the non-reference allele frequency in the RVD2 model to identify rare nucleotide variants in heterogeneous pools.

Our results show that the variational EM algorithm (i) is able to identify rare variants at a 0.1% NRAF level with comparable sensitivity and specificity to a MCMC sampling algorithm; (ii) has a higher specificity in comparison with many state-of-the-art algorithms in a broad range of NRAFs; and (iii) detects SNVs early in the evolutionary time course, as well as tracks NRAF in a real longitudinal yeast data set.

We have chosen parametric forms for the variational distributions. This choice has left us with a complex integral in our variational optimization problem. In future work, we plan to explore other approximations of the variational distributions that render the integral easier to compute. One could use cubic splines to numerically approximate the function and then integrate that surrogate [25]. Another strategy is to consider a Laplace approximation for the variational distribution, as we and others have done previously [26, 27].

Improving the speed of the estimating algorithm enables us to interrogate whole-genome sequencing data. By doing this, we hope to reveal the dynamics of arising variants at the genome-wide scale to show the genetic basis of clonal interference. Our method could be extended to study drug resistance by characterizing tumor heterogeneity in targeted anti-cancer chemotherapy samples, or to find the causative variants that lead to drug resistance and understand the causes of resistance at the single nucleotide level.

## Additional files

Additional file 1 Derivation of the variational expectation maximization (EM) inference algorithm. Derivation of the variational EM algorithm is described in detail. (PDF 46.4 kb)

Additional file 2 Identified variants and corresponding non-reference allele frequencies in gene MTH1 on Chromosome 4. A blank cell indicates that the position of that time point is not called significantly different than G7. The positions highlighted as blue were also identified by Kvitek, 2013. The other 81 positions are novel identified variants in 8 timepoints. (XLSX 18.4 kb)

## Abbreviations

ELBO: Evidence lower bound
EM: Expectation maximization
KL: Kullback-Liebler
MAP: Maximum a-posteriori
MCMC: Markov Chain Monte Carlo
NGS: Next-generation sequencing
NRAF: Non-reference allele frequency
RVD: Rare variant detection
SNVs: Single nucleotide variants
