# Supplementary material for: Variational inference for rare variant detection in deep, heterogeneous next-generation sequencing data
Source: BMC Bioinformatics. 2017 Jan 19;18:45. doi: 10.1186/s12859-016-1451-5 (PMC5244592; doi:10.1186/s12859-016-1451-5)
Supplement: Additional file 1 — Derivation of the variational expectation maximization (EM) inference algorithm. Derivation of the variational EM algorithm is described in detail. (PDF 46.4 kb) [file 12859_2016_1451_MOESM1_ESM.pdf]

Derivation of the Variational Expectation Maximization (EM) Inference Algorithm

1 Evidence Lower Bound

The ELBO can be expanded as

$$\begin{aligned}\mathcal{L}(q,\phi) &= E_q\left[\log p\left(r,\mu,\theta|n;\phi\right)\right] - E_q\left[\log q\left(\mu,\theta\right)\right] \\ &= E_q\left[\log p\left(r|\theta,n\right)\right] + E_q\left[\log p\left(\theta|\mu;M\right)\right] + E_q\left[\log p\left(\mu;\mu_0,M_0\right)\right] \\ &- E_q\left[\log q\left(\mu\right)\right] - E_q\left[\log q\left(\theta\right)\right].\end{aligned} \tag{1}$$

We write out each component below.

$$\begin{aligned}E_q\left[\log p\left(r|\theta,n\right)\right] &= \sum_{j=1}^{J}\sum_{i=1}^{N} E_q\left[\log p\left(r_{ji}|\theta_{ji},n_{ji}\right)\right] \\ &= \sum_{j=1}^{J}\sum_{i=1}^{N} \log\left(\frac{\Gamma(n_{ji}+1)}{\Gamma(r_{ji}+1)\Gamma(n_{ji}-r_{ji}+1)}\right) \\ &+ \sum_{j=1}^{J}\sum_{i=1}^{N}\left\{r_{ji}E_q\left[\log\theta_{ji}\right] + (n_{ji}-r_{ji})E_q\left[\log(1-\theta_{ji})\right]\right\}\end{aligned} \tag{2}$$

$$\begin{aligned}E_q\left[\log p\left(\mu;\mu_0,M_0\right)\right] &= \sum_{j=1}^{J} E_q\left[\log p\left(\mu_j;\mu_0,M_0\right)\right] \\ &= J*\log\frac{\Gamma(M_0)}{\Gamma(\mu_0 M_0)\Gamma(M_0(1-\mu_0))} \\ &+ \sum_{j=1}^{J}\left\{(M_0\mu_0-1)E_q\left[\log\mu_j\right]\right\} \\ &+ \sum_{j=1}^{J}\left\{(M_0(1-\mu_0)-1)E_q\left[\log(1-\mu_j)\right]\right\}\end{aligned} \tag{3}$$

$$\begin{aligned} E_q\left[\log p\left(\theta|\mu;M\right)\right] &= \sum_{j=1}^{J}\sum_{i=1}^{N} E_q\left[\log p\left(\theta_{ji}|\mu_j;M_j\right)\right] \\ &= N * \sum_{j=1}^{J} E_q\left[\log\left(\frac{\Gamma(M_j)}{\Gamma(\mu_j M_j)\Gamma(M_j(1-\mu_j))}\right)\right] \\ &\quad + \sum_{j=1}^{J}\sum_{i=1}^{N}\left\{M_j E_q\left[\mu_j\right] E_q\left[\log\theta_{ji}\right] - E_q\left[\log\theta_{ji}\right]\right\} \\ &\quad + \sum_{j=1}^{J}\sum_{i=1}^{N}\left\{\left(M_j - 1 - M_j E_q\left[\mu_j\right]\right) E_q\left[\log\left(1-\theta_{ji}\right)\right]\right\} \end{aligned} \tag{4}$$

Therefore, we need to compute the following expectations with respect to the variational distribution: $E_q\left[\log\theta_{ji}\right]$, $E_q\left[\log\left(1-\theta_{ji}\right)\right]$, $E_q\left[\log\mu_j\right]$, $E_q\left[\log(1-\mu_j)\right]$, $E_q\left[\mu_j\right]$, and $E_q\left[\log\left(\frac{\Gamma(M_j)}{\Gamma(\mu_j M_j)\Gamma(M_j(1-\mu_j))}\right)\right]$.

We select the functional forms for the variational distributions $q(\theta)$ and $q(\mu)$ to facilitate these expected value computations.

2 Variational Distributions

Since θ and r are conjugate pairs, the posterior distribution of θ_{ji} is a Beta distribution,

$$p(\theta_{ji}|r_{ji}, n_{ji}, \mu_j, M_j) \sim \text{Beta}(r_{ji} + M_j\mu_j, n_{ji} - r_{ji} + M_j(1-\mu_j)). \tag{5}$$

Therefore, we propose a Beta distribution with parameter vector δ_{ji} as variational distribution,

$$\theta_{ji} \sim \text{Beta}(\delta_{ji1}, \delta_{ji2}).$$

The posterior distribution of μ_j is given by its Markov blanket,

$$p(\mu_j|\theta_{ji}, M_j, \mu_0, M_0) \propto p(\mu_j|\mu_0, M_0)p(\theta_{ji}|\mu_j, M_j). \tag{6}$$

This is not in the form of any known distribution. But, since the support of μ_j is $[0, 1]$, we propose a Beta distribution with parameter vector γ_j as variational distribution,

$$\mu_j \sim \text{Beta}(\gamma_{j1}, \gamma_{j2}).$$

Given these variational distributions, we have

$$\begin{aligned} E_q\left[\log\theta_{ji}\right] &= \psi(\delta_{ji1}) - \psi(\delta_{ji1} + \delta_{ji2}) \\ E_q\left[\log\left(1-\theta_{ji}\right)\right] &= \psi(\delta_{ji2}) - \psi(\delta_{ji1} + \delta_{ji2}) \\ E_q\left[\mu_j\right] &= \frac{\gamma_{j1}}{\gamma_{j1} + \gamma_{j2}} \\ E_q\left[\log\mu_j\right] &= \psi(\gamma_{j1}) - \psi(\gamma_{j1} + \gamma_{j2}) \\ E_q\left[\log(1-\mu_j)\right] &= \psi(\gamma_{j2}) - \psi(\gamma_{j1} + \gamma_{j2}), \end{aligned} \tag{7}$$

where ψ is the digamma function.

Since there is no analytical representation for $E_q\left[\log\left(\frac{\Gamma(M_j)}{\Gamma(\mu_j M_j)\Gamma(M_j(1-\mu_j))}\right)\right]$, we must resort to numerical integration,

$$\begin{aligned} &E_q\left[\log\left(\frac{\Gamma(M_j)}{\Gamma(\mu_j M_j)\Gamma((1-\mu_j)M_j)}\right)\right] = \\ &\int_0^1 q(\mu_j;\gamma_{j1},\gamma_{j2})\log\left(\frac{\Gamma(M_j)}{\Gamma(\mu_j M_j)\Gamma((1-\mu_j)M_j)}\right)d\mu_j. \end{aligned} \tag{8}$$

Here $q(\mu_j;\gamma_{j1},\gamma_{j2})$ is the probability density function of the Beta distribution that is calculated using the Python built-in function `scipy.stats.beta.pdf`,

and $\log\left(\frac{\Gamma(M_j)}{\Gamma(\mu_j M_j)\Gamma((1-\mu_j)M_j)}\right)$ is calculated using the Python built-in function

`scipy.special.betaln`. Unfortunately, this numerical integration step is computationally expensive. Finally, the entropy terms can be computed as follows,

$$\begin{aligned} E_q\left[\log q\left(\mu\right)\right] &= \sum_{j=1}^{J} E_q\left[\log q(\mu_j)\right] \\ &= -\sum_{j=1}^{J}\left\{\log(B(\gamma_{j1},\gamma_{j2})) - (\gamma_{j1}-1)\psi(\gamma_{j1})\right\} \\ &\quad + \sum_{j=1}^{J}\left\{-(\gamma_{j2}-1)\psi(\gamma_{j2}) + (\gamma_{j1}+\gamma_{j2}-2)\psi(\gamma_{j1}+\gamma_{j2})\right\}; \end{aligned} \tag{9}$$

and

$$\begin{aligned} E_q\left[\log q\left(\theta\right)\right] &= \sum_{j=1}^{J}\sum_{i=1}^{N} E_q\left[\log q(\theta_{ji})\right] \\ &= -\sum_{j=1}^{J}\sum_{i=1}^{N}\left\{\log(B(\delta_{ji1},\delta_{ji2})) - (\delta_{ji1}-1)\psi(\delta_{ji1})\right\} \\ &\quad + \sum_{j=1}^{J}\sum_{i=1}^{N}\left\{-(\delta_{ji2}-1)\psi(\delta_{ji2}) + (\delta_{ji1}+\delta_{ji2}-2)\psi(\delta_{ji1}+\delta_{ji2})\right\}. \end{aligned} \tag{10}$$

3 Variational EM Algorithm

3.1 (E-step): Updating the variational distributions

The terms in the ELBO that depend on $q(\theta_{ji}|\delta_{ji1}, \delta_{ji2})$ are

$$
\begin{aligned}
\mathcal{L}_{[q(\theta_{ji})]} = & \sum_{j=1}^{J}\sum_{i=1}^{N}\left\{r_{ji}E_q\left[\log\theta_{ji}\right] + (n_{ji}-r_{ji})E_q\left[\log(1-\theta_{ji})\right]\right\} \\
& + \sum_{j=1}^{J}\sum_{i=1}^{N}\left\{M_jE_q\left[\mu_j\right]E_q\left[\log\theta_{ji}\right] - E_q\left[\log\theta_{ji}\right]\right\} \\
& + \sum_{j=1}^{J}\sum_{i=1}^{N}\left\{\left(M_j - 1 - M_jE_q\left[\mu_j\right]\right)E_q\left[\log\left(1-\theta_{ji}\right)\right]\right\} \\
& - \sum_{j=1}^{J}\sum_{i=1}^{N}E_q\left[\log q(\theta_{ji})\right]
\end{aligned} \tag{11}
$$

We update the variational parameters by numerically optimizing

$$
\hat{\delta}_{ji1}, \hat{\delta}_{ji2} = \arg\max_{\delta_{ji1},\delta_{ji2}} \mathcal{L}_{[q(\theta_{ji})]} \tag{12}
$$

subject to the constraints that $\delta_{ji1} \geq 0$ and $\delta_{ji2} \geq 0$ and conditioned on fixed values for the other model and variational parameters using Sequential Least SQuares Programming (SLSQP).

We update the variational distribution $q(\mu_j)$ using the partial ELBO depending on γ_j from each position j (13).

$$
\begin{aligned}
\mathcal{L}_{[q(\mu_j)]} = & N\sum_{j=1}^{J}E_q\left[\log\left(\frac{\Gamma(M_j)}{\Gamma(\mu_jM_j)\Gamma(M_j(1-\mu_j))}\right)\right] \\
& + \sum_{j=1}^{J}\sum_{i=1}^{N}\left\{M_jE_q\left[\mu_j\right]E_q\left[\log\theta_{ji}\right] - E_q\left[\log\theta_{ji}\right]\right\} \\
& + \sum_{j=1}^{J}\sum_{i=1}^{N}\left\{\left(M_j - 1 - M_jE_q\left[\mu_j\right]\right)E_q\left[\log\left(1-\theta_{ji}\right)\right]\right\} \\
& + J\log\frac{\Gamma(M_0)}{\Gamma(\mu_0M_0)\Gamma(M_0(1-\mu_0))} \\
& + \sum_{j=1}^{J}\left\{(M_0\mu_0 - 1)E_q\left[\log\mu_j\right] + (M_0(1-\mu_0)-1)E_q\left[\log(1-\mu_j)\right]\right\} \\
& - \sum_{j=1}^{J}E_q\left[\log q(\mu_j)\right]
\end{aligned} \tag{13}
$$

Again, we update the variational parameters by numerically optimizing

$$
\hat{\gamma}_{j1}, \hat{\gamma}_{j2} = \arg\max_{\gamma_{j1},\gamma_{j2}} \mathcal{L}_{[q(\mu_j)]} \tag{14}
$$

subject to the constraints that $\gamma_{j1} \geq 0$ and $\gamma_{j2} \geq 0$ and conditioned on fixed values for the other model and variational parameters using SLSQP. The computational cost of optimizing (13) is high because of the quadrature of $E_q\left[\log\left(\frac{\Gamma(M_j)}{\Gamma(\mu_jM_j)\Gamma(M_j(1-\mu_j))}\right)\right]$ in (8).

3.2 (M-step): Updating the model parameters

We can write out the ELBO as a function of each model parameter μ_0, M_0, and M_j as follows.

The ELBO with respect to μ_0 is

$$\mathcal{L}_{[\mu_0]} = -J * \log\Gamma(\mu_0 M_0) - J * \log\Gamma(M_0(1-\mu_0)) + M_0\mu_0 \sum_{j=1}^{J} \left\{E_q\left[\log\mu_j\right] - E_q\left[\log(1-\mu_j)\right]\right\}. \tag{15}$$

The ELBO with respect to M_0 is

$$\mathcal{L}_{[M_0]} = J * \log\frac{\Gamma(M_0)}{\Gamma(\mu_0 M_0)\Gamma(M_0(1-\mu_0))} + M_0 \sum_{j=1}^{J} \left\{\mu_0 E_q\left[\log\mu_j\right] + (1-\mu_0)E_q\left[\log(1-\mu_j)\right]\right\}. \tag{16}$$

The ELBO with respect to M_j is

$$\mathcal{L}_{[M_j]} = N * \sum_{j=1}^{J} E_q\left[\log\left(\frac{\Gamma(M_j)}{\Gamma(\mu_j M_j)\Gamma(M_j(1-\mu_j))}\right)\right] + M_j \sum_{j=1}^{J}\sum_{i=1}^{N} \left\{E_q\left[\mu_j\right] E_q\left[\log\theta_{ji}\right] + \left(1 - E_q\left[\mu_j\right]\right) E_q\left[\log\left(1-\theta_{ji}\right)\right]\right\}. \tag{17}$$

We also use SLSQP to optimize the ELBO function with respect to each parameter, μ_0, M_0, and M_j. It is computationally easy to optimize μ_0 (15) and M_0 (16). However, it is costly for optimizing M_j (17) because the quadrature is needed to calculate $E_q\left[\log\left(\frac{\Gamma(M_j)}{\Gamma(\mu_j M_j)\Gamma(M_j(1-\mu_j))}\right)\right]$ using (8).
